# Evidenzbasierte Prävention für die psychische Gesundheit von Kindern und Jugendlichen: Der Ansatz „Communities That Care“ (CTC) für Deutschland

**DOI:** 10.1007/s00103-023-03725-0

**Published:** 2023-06-14

**Authors:** Ulla Walter, Frederick Groeger-Roth, Dominik Röding

**Affiliations:** 1 Institut für Epidemiologie, Sozialmedizin und Gesundheitssystemforschung, Medizinische Hochschule Hannover, 30625 Hannover, Deutschland; 2 Landespräventionsrat Niedersachsen, Hannover, Deutschland

**Keywords:** Gesundheitsförderung, Intersektorale Kooperation, Kommune, Systemische Intervention, Risiken und Schutzfaktoren, Health promotion, Intersectoral coalition, Community, Systemic intervention, Risk factors and protective factors

## Abstract

Intersektorale Zusammenarbeit, Evidenzbasierung und nachhaltige Implementation sind zentrale Herausforderungen in der kommunalen Gesundheitsförderung. Diese adressiert das internationale Präventionssystem „Communities That Care“ (CTC). CTC zielt mit einer systemischen Mehr-Ebenen-Strategie auf die Prävention von Alkohol- und Drogenmissbrauch, Gewalt, Delinquenz, Schulabbruch und depressiven Symptomen bei Heranwachsenden. Das in den USA entwickelte, evidenzbasierte und kosteneffektive Präventionssystem wurde nach Deutschland transferiert; die Kosteneffektivität wird derzeit in einer Replikationsstudie überprüft.

CTC ist empirisch-theoretisch basiert und folgt einem 5‑phasigen Prozessmodell. Wesentlich für die Akzeptanz und evidenzbasierte Durchführung ist die Bildung einer intersektoralen Koalition, deren Mitglieder mehrjährig beratend begleitet und geschult werden. Die Akteur:innen werden befähigt, auf kommunaler Ebene ein Systemveränderungsmodell einzusetzen und langfristig zu implementieren. Ziel ist es, evidenzbasierte Maßnahmen datenbasiert und bedarfsorientiert auszuwählen und unter Berücksichtigung der Kontextbedingungen vor Ort zu implementieren, um Risikofaktoren zu reduzieren, Schutzfaktoren zu fördern und damit die Gesundheit der Heranwachsenden zu verbessern. Validierte Instrumente wie der CTC-Kinder- und Jugendsurvey sowie das Register „Grüne Liste Prävention“ mit evidenzbasierten Programmen unterstützen den Prozess.

CTC integriert als systemische Intervention vorhandene örtliche Strukturen und Organisationen und bindet diese über neue Entscheidungs- und Entwicklungsgremien in den gesamten Prozess ein. Auf diese Weise kann das Potenzial in der Kommune so gut wie möglich genutzt, Ressourcen gebündelt, Kräfte entfaltet und Transparenz hergestellt werden.

## Abstract (en)

Intersectoral collaboration, evidence base, and sustainable implementation are central challenges in community health promotion. The international prevention system Communities That Care (CTC) addresses these challenges. CTC aims to prevent alcohol and drug abuse, violence, delinquency, school dropout, and depressive symptoms among adolescents with a systemic multi-level strategy. The evidence-based and cost-effective prevention system developed in the USA was adapted to Germany; at present, a replication study evaluates the cost-effectiveness.

CTC is based on empirical theory and follows a five-phase process model. Essential for acceptance and evidence-based implementation is the formation of an intersectoral coalition, whose members receive advisory support and training over several years. The actors are empowered to use a system change model at the municipal level and to implement it in the long term. The aim is to select evidence-based measures in a data-driven and needs-oriented manner and to implement them in consideration of the local contextual conditions in order to reduce risk factors, promote protective factors, and thus improve the health of adolescents. Validated instruments such as the CTC Children and Youth Survey and a registry with evidence-based prevention programs (“Grüne Liste Prävention”) support the process.

As a systemic intervention, CTC integrates existing local structures and agencies and involves them in the entire process through new decision-making and development bodies. In this way, the potential in the municipality is used and, as much as possible, resources are bundled, strengths are developed, and transparency is created.

## Einleitung: Herausforderungen

Das Gesundheitsziel „gesund aufwachsen“ unterstreicht die Relevanz der psychischen Gesundheit sowie der systematischen Prävention psychischer Beeinträchtigungen und der Gesundheitsförderung in der ersten Lebensphase. Mit dem Präventionsgesetz und der Nationalen Präventionsstrategie wird die Förderung der psychischen Gesundheit auf kommunaler Ebene gestärkt [1]. Die Ausgestaltung ist bislang unkoordiniert, geprägt von einer unzureichenden Nutzung vorhandener Gestaltungshilfen [2–4] und erheblichen Lücken bezüglich der Evidenz der kommunalen Ansätze [5, 6]. Mehr-Ebenen-Strategien und systemische Transformationen haben das Potenzial, Kompetenzen zu stärken, Maßnahmen alltagsintegriert zu adressieren und Ressourcen zu bündeln [7]. Solch komplexe Interventionen erfordern eine sorgsame Planung und abgestimmte Umsetzung, um die vielfältigen strukturellen sowie fachlich-methodischen Herausforderungen zu meistern.

### Herausforderung 1: Sektorengrenzen überwinden.

Die psychische Gesundheit von Heranwachsenden tangiert mit ihren Symptomen der Schulabstinenz, Gewalterfahrung, Depressionen und Ängsten sowie Sucht sowohl die Bereiche Gesundheit, Soziales und Bildung als auch die Kriminalprävention. Trotz vielfältiger Schnittmengen bezüglich Ziele und Vorgehensweisen erfolgt eine Zusammenarbeit in der Kommune bislang nur partiell [2, 3]. Dieses ist bedingt durch Unterschiede im Fachverständnis, rechtliche Regelungen und gewachsene Strukturen [8].

*Intersektorale Netzwerkarbeit* und die Einbindung vielfältiger (Schlüssel‑)Akteur:innen gelten als Erfolgsfaktoren zur Erschließung von Handlungsfeldern, Daten auf kommunaler Ebene und Ressourcen [6]. Die intersektorale Netzwerkarbeit stellt ein dynamisches System dar, das einem längeren Prozess unterliegt, der auf eine Transformation von organisationalen Kulturen, Praktiken, aber auch des Verständnisses von Gesundheit und ihrer Förderung zielt. Wesentlich für erfolgreiche, langfristige intersektorale Kooperationen ist Capacity Building (z. B. gemeinsame Mission, Partizipation, modell-/theoriegeleitete Strategien und transparente Kommunikation; [9]).

### Herausforderung 2: Evidenzbasiert vorgehen.

Evidenzbasierung gilt als zentral, um Prävention und Gesundheitsförderung (PGF) dauerhaft als starke 4. Säule im Gesundheitssystem zu etablieren [10]. Bislang bleibt in Deutschland das Potenzial hierzu allerdings weitgehend ungenutzt. In der Praxis besteht nur unzureichendes Wissen über den Nutzen von Evidenzbasierung und (inter)national vorliegenden Erkenntnissen; die Relevanz evidenzbasierter Empfehlungen ist oft noch umstritten und wird als kollidierend mit grundlegenden Prinzipien der Gesundheitsförderung wie der Partizipation wahrgenommen [11, 12]. Zur Förderung evidenzbasierter (oder besser: evidenzinformierter) Entscheidungen ist eine praxis- und politikfreundliche Aufbereitung der Erkenntnisse zur Entstehung von (psychischer) Gesundheit, Veränderungstheorien und vorliegender Evidenz erforderlich [10, 11]. Um Akteur:innen systematisch bei der Auswahl evidenzbasierter Programme zu unterstützen, empfiehlt das „Memorandum Evidenzbasierte Prävention und Gesundheitsförderung“ der Bundeszentrale für gesundheitliche Aufklärung (BZgA; [10]) eine Datenbank mit systematisch aufbereiteten Wirksamkeitsnachweisen.

In Deutschland liegen inzwischen zahlreiche evaluierte, überwiegend verhaltensbezogene Programme vor [13, 14], wobei erwartungsgemäß Effekte bei universellen Programmen im kleinen bis moderaten Bereich liegen [13]. Für ihre Wirksamkeit ist die Implementationsqualität besonders wichtig. Häufig vorgenommene pragmatische Veränderungen [15] sind zu vermeiden, effektiver sind reflektierte kultursensible Anpassungen [16].

### Herausforderung 3: Nachhaltig verankern.

Soll PGF als starke 4. Säule im Gesundheitssystem etabliert werden, sind zudem ihre systemische Verankerung in der Kommune sowie eine kontinuierliche Qualitätsentwicklung der ergriffenen Maßnahmen notwendig [17, 18]. Dieses erfordert nicht nur einen Konsens aller Beteiligten, sondern auch deren Qualifizierung und instrumentelle Unterstützung.

Das in den USA entwickelte, weltweit verbreitete (USA, Kanada, Deutschland, Schweden, Schweiz, Niederlande, Großbritannien, Zypern, Kroatien, Australien, Kolumbien, Chile) und nach Deutschland transferierte Präventionssystem „Communities That Care“ (CTC) adressiert alle 3 Herausforderungen. Es setzt am Dachsetting Kommune an und bezieht weitere Lebenswelten sowie Versorgungsbereiche ein.

Ziele des Beitrages sind, (1) einen Überblick über das Konzept des Präventionssystems CTC mit seinen zentralen Elementen zu geben, (2) die bestehende Evidenz darzulegen und (3) die Entwicklung in Deutschland aufzuzeigen.

## Das Präventionssystem (CTC)

### Die Entwicklung in den USA

Das in den USA von einer interdisziplinären Forschungsgruppe entwickelte CTC-Konzept geht auf die frühen 1980er-Jahre zurück [19, 20]. Ziel war es zunächst, einen wirksamen Ansatz zur Prävention von Substanzmissbrauch, Gewalt und Delinquenz bei Heranwachsenden theoretisch-empirisch fundiert zu konzipieren [21]. Ab Ende der 1980er-Jahre verband sich die Entwicklung des CTC-Präventionssystems mit den Ideen der Gesundheitsförderung, allem voran [22]: (1) die Verknüpfung verhaltens- und verhältnisbezogener Interventionen, (2) die Adressierung von Risiko- (RF) und Schutzfaktoren (SF) sowie (3) die Befähigung (Empowerment) von Kommunen, ihren lokalen Bedarf an PGF objektiv zu identifizieren und wirksam zu beeinflussen. In den 1990er-Jahren wurde CTC in einem partizipativen Prozess gemeinsam mit 472 Kommunen in den USA weiterentwickelt und an über 500 Standorten getestet [22]. Während dieser Phase mehrte sich die Evidenz, dass die gesundheitsbezogene Wirksamkeit kommunaler Gesundheitsförderung davon abhängt, dass spezifische lokale Bedarfe adressiert werden und die präventiven bzw. gesundheitsfördernden Maßnahmen kommunales Gemeinschaftseigentum sind [23].

Kern der Weiterentwicklung von CTC war die Überwindung von 2 verbreiteten Problemen [24]: (1) der Einsatz von Maßnahmen ohne ausreichende wissenschaftliche Evidenz, (2) die unzulängliche Implementation von nachweislich effektiven Maßnahmen. Auch zeigten viele Studien, dass partizipativ aufgebaute kommunale Netzwerke für PGF nicht per se zu einer effektiven kommunalen Gesundheitsförderung führen [24].

Die Entwickler:innen von CTC nahmen deshalb die als Erfolgsfaktoren für wirksame intersektorale Kooperationen auf lokaler Ebene (Community Coalitions) geltenden Aspekte in das CTC-System mit auf [24]: (1) klar definierte, messbare Ziele und Outcomes, (2) Aufbau qualitativ hochwertiger Datenquellen und eines Monitoring-Systems, (3) Einsatz evidenzbasierter Programme verbunden mit einem Monitoring und Qualitätsmanagement sowie (4) Evaluierung der implementierten Programme anhand aussagekräftiger Outcomes.

Kern des CTC-Präventionssystems [24, 25] sind die Zusammenführung und Qualifizierung kommunaler Entscheidungsträger sowie der Auf- bzw. Ausbau eines kommunalen Kooperationsnetzwerkes für PGF. Diese systemische Intervention (Abschnitt „Die systemische Intervention auf kommunaler Ebene“) basiert auf 5 Phasen mit jeweils eigenen Benchmarks und Meilensteinen, die die Kommune dabei unterstützen, ihren Implementationsfortschritt zu monitoren und zu lenken (Details: [26]).

### Die theoretisch-empirische Basis

CTC basiert von Beginn an auf theoretischen Konzepten und empirischen Erkenntnissen, die im Folgenden skizziert werden.

Das* Konzept der RF und SF* bildet die zentrale Grundlage. Belastungen und Ressourcen in Kindheit und Jugend bestimmen die Entwicklung Heranwachsender, die Ausbildung von gesundheitsrelevantem Verhalten und ihre (psychische) Gesundheit wesentlich mit. Die individuelle Resilienz steht dabei im engen Zusammenhang mit kontextbezogenen Faktoren (Familie, Peers, Schule, Sozialraum; [27–29]). Die Faktoren zeigen erhebliche Überschneidungen ihrer Wirkungen, sie wirken übergreifend auf eine Vielzahl von psychosozialen Problemen und Verhaltensweisen [30, 31]. Dabei unterscheiden sich Risikoprofile kleinräumig in Städten, Gemeinden etc. [32]. Dieses ermöglicht eine gezielte Adressierung der besonderen Belastungen sowie die Förderung der Ressourcen und gleichzeitig eine Prävention in mehreren Bereichen.

**Figure 1:**
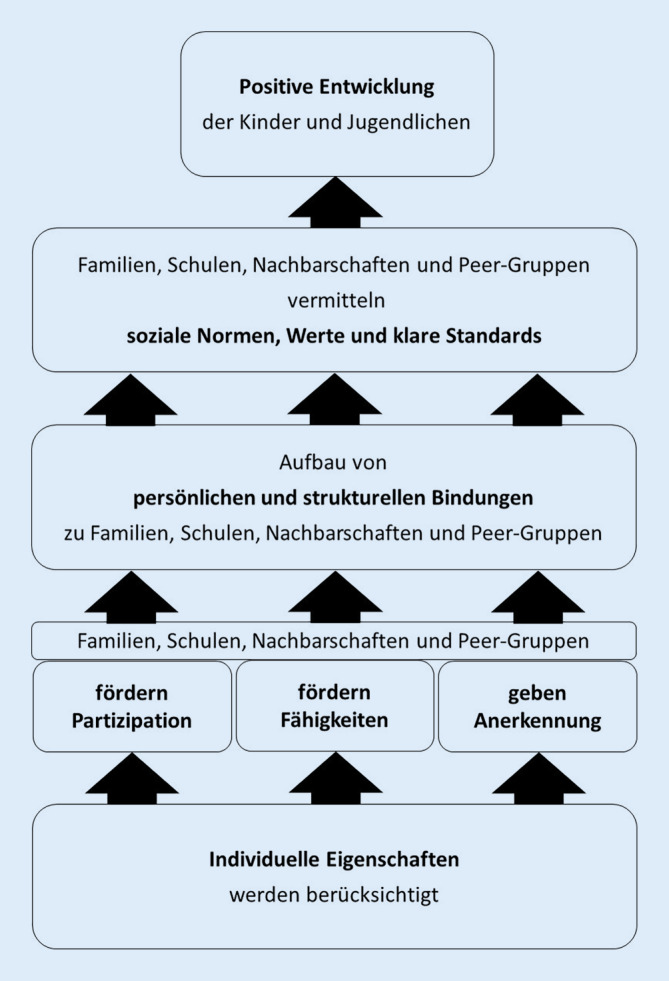

Das *Modell der sozialen Entwicklung* (Social Development Model [SDM]) als entwicklungstheoretische Basis des konzeptionellen CTC-Ansatzes basiert auf den SF (Abb. 1; [33, 34]). Ihre Stärkung in den oben genannten Bereichen – z. B. über Partizipation der Beteiligten, Förderung von Kompetenzen und einer Anerkennungskultur – unterstützt die Erreichung des Ziels, das gesundheitszuträgliche Verhalten der Kinder und Jugendlichen zu fördern. Dies geschieht über die Intensivierung der Bindungen zu Bezugspersonen und Settings, die eine Vorbildfunktion für das Verhalten haben.

**Figure 2:**
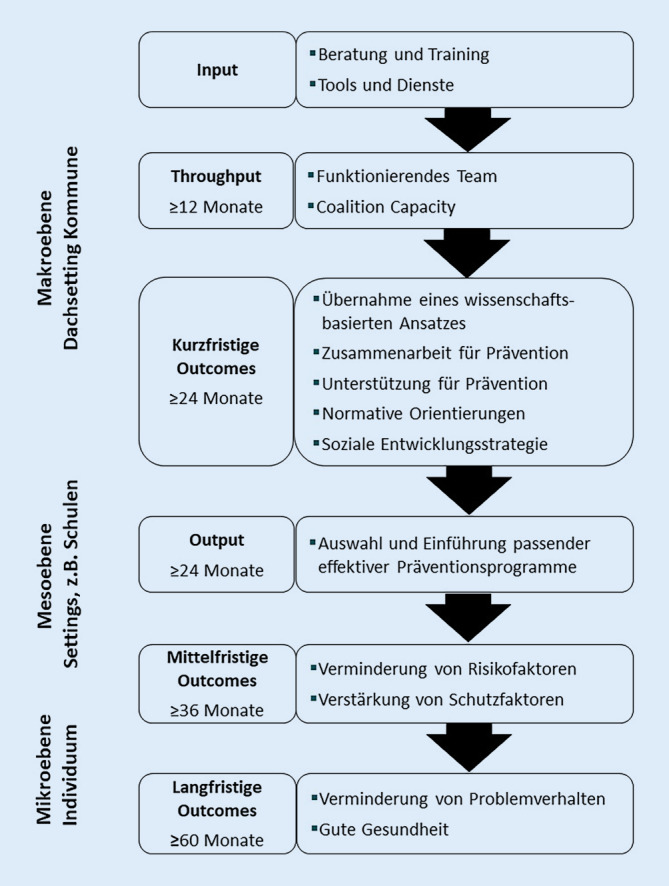

Die *CTC-Theory of Change* bildet das gesamte CTC-Präventionssystem sowie die zu erwartenden kurz-, mittel- und langfristigen Ergebnisse auf der Makro‑, Meso- und Mikroebene in einem logischen Modell ab (Abb. 2). Systemische Interventionen wie intersektorale Kooperationen werden dabei selbst zu einem Ansatz der PGF auf der Makro- und Mesoebene, um insbesondere auf der Meso- und Mikroebene Veränderungen zu erreichen.

Angenommen wird, dass (1) die edukativen Maßnahmen (Schulungen, Prozessberatung) die Kompetenzen der Beteiligten und ihre Zusammenarbeit fördern sowie den Aufbau von Strukturen und Ressourcen unterstützen (Coalition Capacity); (2) die aufgebauten Koalitionen eine Systemtransformation in der Kommune bewirken (vermehrte Wissenschaftsbasierung und interorganisationale Zusammenarbeit, Wandel der gesundheitsbezogenen Normen, Etablierung einer sozialen Entwicklungsstrategie); (3) die Kommunen die empirisch ermittelten spezifischen lokalen Bedarfe mit evidenzbasierten Maßnahmen gezielt adressieren und diesen Prozess qualitätsgesichert steuern; (4) bei den Kindern und Jugendlichen die kontextbezogenen RF reduziert und die SF erhöht werden, Problemverhalten sich zugunsten gesundheitszuträglichen Verhaltens verändert und sich letztlich die (psychische) Gesundheit der Heranwachsenden verbessert.

### Wirksamkeit

Die US-amerikanische *Community Youth Development Study* (CYDS) untersuchte die (Kosten‑)Effektivität von CTC und überprüft diese weiterhin in einer Langzeitstudie. In diese randomisierte, kontrollierte Studie (RCT) sind 24 Kommunen aus 7 US-Bundesstaaten einbezogen [24]. Inzwischen liegen zahlreiche Publikationen zu den kurz-, mittel- und langfristigen Effekten auf unterschiedlichen Ebenen vor. Grundlage bildet jeweils das logische Modell (Abb. 2).

Auf der *Makroebene* nahm der Grad wissenschaftsbasierter PGF deutlich zu. Die Wahrscheinlichkeit, die höchste Stufe im Adoption Score (Grad der Übernahme eines wissenschaftsbasierten Präventionsansatzes) zu erreichen, ist für die CTC-Kommunen gegenüber den Kontrollkommunen über 5‑mal so hoch (Odds Ratio [OR] = 5,37; [35]). Dieser Effekt war auch noch 5 Jahre später nachweisbar (OR = 4,0; 95 %-Konfidenzintervall, KI: 2,51–5,49; [26]). Er ist abhängig vom Erfolg des Coalition Capacity Building [36] und wird mediiert durch die Aneignung neuer Kompetenzen im Bereich PGF sowie die interorganisationale Vernetzung. Die bereits hohe Implementierungsqualität der evidenzbasierten Maßnahmen konnte über Jahre gehalten werden und zeigte sich auch noch 2 Jahre nach Studienende [37].

Auf der *Mesoebene* waren erste Effekte knapp 3 bzw. 1,67 Jahre nach bedarfsorientierter Adressierung der evidenzbasierten Maßnahmen sichtbar [38]. Nach einem multivariaten Modell sanken in den CTC-Kommunen die von ihnen jeweils priorisierten RF im Mittel leicht (z. B. geringe Bindung an die Schule, Freunde mit jugendlichem Problemverhalten, Konflikte in der Familie, rebellische Einstellungen sowie soziale Normen im Wohnumfeld, die Alkohol- und Drogenkonsum von Heranwachsenden billigen).

Auf der *Mikroebene* zeigten die Kinder und Jugendlichen außerhalb von CTC-Kommunen im Vergleich zu denen in CTC-Kommunen höhere Inzidenzen für erstmaligen Alkoholkonsum (OR = 1,60; *p* < 0,05), für erstmaliges Zigarettenrauchen (OR = 1,79; *p* < 0,05) und erstmalig delinquentes Verhalten (OR = 1,41; *p* < 0,05) sowie erstmaligen Konsum von Kau- und Schnupftabak (OR = 2,34; *p* < 0,01; [39]). Auch wiesen die Heranwachsenden außerhalb von CTC-Kommunen höhere Prävalenzen auf für Alkoholkonsum (OR = 1,25; 95 %-KI: 1,04–1,52), Konsum von Kau- und Schnupftabak (OR = 1,79; 95 %-KI: 1,23–2,62), Rauschtrinken (OR = 1,40; 95 %-KI: 1,07–1,84) und delinquentes Verhalten (OR = 1,34; 95 %-KI: 1,20–1,49; [39]). Langzeiteffekte zeigten sich noch, als die Kohorte der Fünftklässler das 21. Lebensjahr erreicht hatte [22].

CTC zeigt damit neben der nur in den USA angewandten Intervention PROSPER [40] als einzige RCT-Studien Effekte auf Kommunalebene. Die geringere Prävalenz problematischen Verhaltens bei den CTC-Schüler:innen ist nach einer Mediationsanalyse zu 96 % auf die stärkere Übernahme eines wissenschaftsbasierten PGF-Ansatzes zurückzuführen [41].

Ökonomische Evaluationen zeigen, dass der CTC-Ansatz auch monetär eine gute Investition ist. Bei Implementationskosten von 556 USD/heranwachsender Person beträgt der Nettonutzen nach 5 Jahren 3920 $/heranwachsender Person, womit eine Kosten-Nutzen-Relation von 1:8,22 erreicht wird [42]. Langzeitanalysen 12 Jahre nach der Baseline und bei einem Alter der Heranwachsenden von 23 Jahren zeigen bei Betrachtung der primären Outcomes (Alkohol, Drogen, antisoziales Verhalten) eine Kosten-Nutzen-Relation von 1:12,88. Werden zusätzlich sekundäre Outcomes wie Schulabschluss einbezogen, beträgt die Kosten-Nutzen-Relation 1:30,62 [43].

## Etablierung und Entwicklungen in Deutschland

### Übertragung und Anpassung der CTC-Elemente

Der CTC-Ansatz wurde von 2009 bis 2012 vom Landespräventionsrat Niedersachsen (LPR) im Rahmen eines EU-Modellversuchs an den deutschen Kontext angepasst. Dieses beinhaltet (1) das systemische Interventionskonzept auf kommunaler Ebene (Fazit und Ausblick), (2) den CTC-Kinder- und Jugendsurvey sowie (3) das Evidenzregister „Grüne Liste Prävention“ (www.gruene-liste-praevention.de).

Zur Präventionsplanung und -steuerung wurde der CTC-Kinder- und Jugendsurvey entwickelt und in Niedersachen erprobt (siehe unten). Die Transferabilität hinsichtlich seiner methodischen Validität wurde anhand einer repräsentativen Stichprobe überprüft [44]. Der Survey erwies sich aus praktischer Sicht als übertragbar, methodisch wurden Modifizierungen vorgenommen.

Ein Evidenzregister wie das US-amerikanische Vorbild, die „Blueprints for Health Youth Development“ (www.blueprintsprograms.org), bestand in Deutschland nicht. Um die Akteur:innen systematisch bei der Auswahl evidenzbasierter Programme zu unterstützen, entwickelte der LPR 2011 die Grüne Liste Prävention [14]. Dieses Evidenzregister wird seitdem kontinuierlich weiterentwickelt und aktualisiert. Einen Überblick über die Struktur und Inhalte gibt die Infobox 1.

Nach einer Machbarkeitsstudie [45] ist CTC seit 2013 in Niedersachsen regelhaft implementiert [12]. Über die 2018 von der Stiftung Deutsches Forum für Kriminalprävention (DFK) und der Deutschen Präventionstag gGmbH (DTP) gegründeten Transferstelle CTC wird die Implementation in weiteren Bundesländern unterstützt. Das CTC-Konzept richtet sich in Deutschland an Kommunen unterschiedlicher Größe und Struktur (Landkreis, Stadt, Stadtteil). Voraussetzung für die Teilnahme ist ein Stadt- bzw. Gemeinderatsbeschluss, um eine breite Akzeptanz und Bereitschaft vor Ort zu gewährleisten. Inzwischen weisen in 5 Bundesländern über 50 Kommunen Erfahrungen mit diesem Konzept auf (Stand: 01/2023).

**Figure 3:**
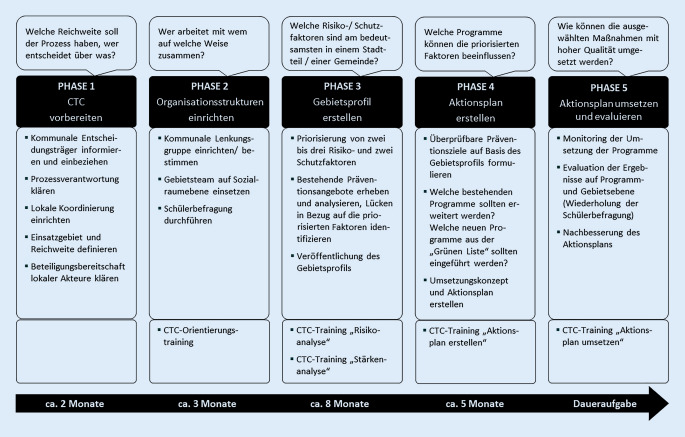

Entsprechend dem US-amerikanischen Original liegen dem CTC-System in Deutschland das Modell der sozialen Entwicklung (Abb. 1), das logische Modell (Abb. 2) sowie die 5‑phasige Intervention (Abb. 3) zugrunde. CTC erfüllt die Kriterien der Evidenzbasierung (Infobox 2).

Die Adaption erfolgte vor allem strukturell und kulturell. So baut das CTC-Konzept in Deutschland im Gegensatz zu den USA i. d. R. auf bestehende Strukturen und Netzwerke auf und kann diese personell und strukturell erweitern. Während in den USA Ehrenamtliche in den kommunalen Koalitionen dominieren, wird in Deutschland vorrangig mit den vorhandenen professionellen Strukturen gearbeitet. Die begleitenden Schulungen wurden entsprechend angepasst sowie die CTC-Instrumente sprachlich und kulturell adaptiert.

### Bedarfsanalyse und Evaluation: der CTC-Kinder- und Jugendsurvey

Ein wesentlicher Bestandteil des CTC-Systems ist der Kinder- und Jugendsurvey. Die Online-Befragung berücksichtigt die Handlungsfelder Gewalt, Delinquenz, Alkohol- und Drogenmissbrauch, Schulabbruch, Teenagerschwangerschaft sowie depressive Symptomatik und Wohlbefinden. Außerdem werden RF und SF für 4 Bereiche erfasst, die hier beispielhaft illustriert werden [44]:

- *Familie *(RF: Probleme mit dem Familienmanagement, Zustimmung der Eltern zu antisozialem Verhalten; SF: familiärer Zusammenhalt, familiäre Gelegenheiten zur prosozialen Mitwirkung),
- *Schule* (RF: Lernrückstände, fehlende Bindung zur Schule; SF: schulische Gelegenheiten und Anerkennung für prosoziale Mitwirkung),
- *Kinder und Jugendliche* (RF: Entfremdung und Auflehnung, Umgang mit Freunden, die antisoziales Verhalten/Substanzkonsum zeigen; SF: moralische Überzeugungen und klare Normen, Interaktion mit prosozialen Peers),
- *Nachbarschaft/Wohngegend* (RF: soziale Desorganisation und fehlende Bindung zur Nachbarschaft, wahrgenommene Verfügbarkeit von Alkohol/Tabak/Drogen/Waffen; SF: Gelegenheiten und Anerkennung für prosoziale Mitwirkung).

In Niedersachsen wird seit 2013 2‑jährlich eine landesweite Schüler:innenbefragung durchgeführt. Die Daten dienen in diesem Bundesland als *Referenzwerte* für kommunale Erhebungen und ermöglichen damit Aussagen zum lokalen PGF-Bedarf. Die landesweite Stichprobe ist repräsentativ bezüglich der Jahrgangsstufen 6 bis 11, Schulformen und städtischen/ländlichen Gebiete. Die Befragung erfolgte 2017 in 308 Klassen an 268 Schulen, anvisiert wurden 7000 Schüler:innen. Die Beteiligungsrate lag bei 39,3 % [46]. Inzwischen wird in Niedersachsen zusätzlich eine Grundschulvariante der CTC-Befragung getestet.

### Die systemische Intervention auf kommunaler Ebene

Das CTC-Präventionssystem besteht aus 5 Phasen, die dem Public Health Action Cycle (Problembestimmung, Strategieformulierung, Umsetzung, Bewertung) bzw. dem Plan-Do-Check-Act(PDCA)-Zyklus entsprechen. Über die gesamte Zeit, die über 2 Jahre betragen kann, werden die Beteiligten in 5 Schulungseinheiten qualifiziert und beratend begleitet. Spezifische Instrumente mit definierten Zielen, Meilensteinen und Benchmarks dienen der Qualitätssicherung sowie -entwicklung und unterstützen den Prozess (Abb. 3).

Auf Basis der entwickelten Materialien, Schulungen, Surveys und beratender Unterstützung werden die Akteur:innen befähigt, auf kommunaler Ebene theoretisch und empirisch basiert ein Systemveränderungsmodell einzusetzen und langfristig zu implementieren. Ziel ist es, evidenzbasierte Maßnahmen datenbasiert und bedarfsorientiert auszuwählen und unter Berücksichtigung der Kontextbedingungen vor Ort zu implementieren, um RF zu reduzieren, SF zu fördern und damit die Gesundheit der Heranwachsenden zu verbessern. CTC integriert als systemische Intervention vorhandene örtliche Strukturen und Organisationen und bindet diese über neue Entscheidungs- und Entwicklungsgremien ebenso wie die Bürger:innen in den gesamten Prozess ein. Auf diese Weise kann das Potenzial in der Kommune so gut wie möglich genutzt, Ressourcen gebündelt, Kräfte entfaltet und Transparenz hergestellt werden.

Einen Überblick über die nachfolgend erläuterten 5 Phasen gibt Abb. 3.

#### Phase 1: CTC vorbereiten.

Zunächst werden die strukturellen und personellen Voraussetzungen für die Etablierung von CTC in der Kommune geschaffen. Hierzu zählen die Etablierung einer Kerngruppe von Entscheidungsträger:innen zentraler Einrichtungen, die Identifikation einer Schlüsselperson zur Förderung des Gesamtprozesses in der Kommune sowie die Festlegung der CTC-Koordination. Dabei können bestehende Netzwerke genutzt und ggf. erweitert werden. Zudem müssen die erforderlichen Rahmenbedingungen geklärt und festgelegt werden.

#### Phase 2: CTC einführen und Rückhalt für CTC schaffen.

Eine Befragung der Kinder und Jugendlichen zur Analyse der Ausgangslage und der Präventionsbedarfe (hohe RF, niedrige SF, siehe Phase 3) wird durchgeführt und extern ausgewertet. Eine Lenkungsgruppe wird gegründet, in der eine einflussreiche Person (i. d. R. Bürgermeister:in, Landrät:in) den Vorsitz übernimmt. In dem zu gründenden Gebietsteam sollen die wichtigsten örtlichen Einrichtungen und Expert:innen im Kontext der Kinder- und Jugendgesundheit sowie der PGF vertreten sein.

#### Phase 3: Ein CTC-Gebietsprofil erstellen.

Im Zentrum stehen die Identifikation und Priorisierung der zentralen RF/SF sowie Problemverhaltensweisen. Grundlage bilden neben dem Kinder- und Jugendsurvey regionale Daten der Gesundheitsberichterstattung, z. B. Hilfe zur Erziehung (HzE-Daten), Sozialstrukturdaten (z. B. Arbeitslosengeld-II-Bezug) sowie die polizeiliche Kriminalstatistik (PKS). Zudem werden vorhandene PGF-Angebote analysiert, um bestehende Strukturen und Expertisen zu nutzen, Lücken zu schließen und Überschneidungen zu vermeiden.

#### Phase 4: Einen CTC-Aktionsplan erstellen.

Die Beteiligten definieren überprüfbare Ziele bezüglich des zu adressierenden Problemverhaltens, der Stärkung der SF und Abschwächung der RF. Hierzu werden bedarfsgerecht evidenzbasierte Programme auf Basis der Grünen Liste Prävention (s. Infobox 1) ausgewählt und ein Konzept erstellt, wie vorhandene Angebote mit neu ausgewählten verknüpft werden können. Zentral ist, dass die Lenkungsgruppe dem Aktionsplan zustimmt, dieser somit kommunal getragen wird und die erforderlichen Ressourcen zur Verfügung stehen.

#### Phase 5: Einen CTC-Aktionsplan ausführen.

Die ausgewählten Maßnahmen werden nun implementiert und durchgeführt. Hierzu wird die erforderliche Organisationsstruktur überprüft, ggf. weitere Einrichtungen hinzugenommen und Kooperationsvereinbarungen getroffen. Diese Phase ist zeitlich unbegrenzt. Um Veränderungen bei den von der Kommune priorisierten RF/SF auf Verhaltensebene zu messen, wird die Befragung der Kinder und Jugendlichen alle 2 bis 3 Jahre wiederholt. Sie bildet zugleich eine Basis für die Justierung des Aktionsplans.

Kommunen unterscheiden sich stark bezüglich der Ausgangs- und Rahmenbedingungen für Konzepte wie CTC. Eine wichtige Rahmenbedingung ist die Größe der Kommune bzw. die sich aus der Größe ergebende Komplexität der kommunalen Akteur:innenlandschaft. Während in Niedersachsen CTC vor allem im ländlichen Raum in Landkreisen und kleineren Kommunen umgesetzt wird, liegen mittlerweile auch positive Erfahrungen in größeren Kommunen vor, wie z. B. in Augsburg^1^. Hier wurde nach Rückmeldung aus der Kommune durch den Einsatz von CTC z. B. eine verbesserte Kooperation der Fachplanungen in der Kommunalverwaltung in den Bereichen Jugend, Schule, Stadtplanung und Gesundheit erreicht.

## Fazit und Ausblick

Verhältnisorientierte sowie kombinierte verhaltens- und verhältnisbezogene Interventionen werden trotz anerkannter Bedeutung seltener implementiert und evaluiert – unter anderem aufgrund ihrer Komplexität und mangels adäquater Studiendesigns. Dieses trifft auch für die kommunale PGF mit ihrer systemischen Steuerung der intersektoralen Koalitionen zu. Gerade bei komplexen und längerfristig wirkenden Interventionen werden nur selten Veränderungen der (psychischen) Gesundheit bei den eigentlichen Endadressat:innen erhoben, eine Verknüpfung von Output- und Outcome-Parametern sowie von Primär- und Sekundärdaten fehlt häufig.

Hier setzt das in den USA entwickelte Präventionssystem CTC an. Bei sorgsamer Implementation und intensiver Begleitung der Kommunen zeigen sich auf allen Ebenen auch langfristig positive Effekte.

Bei CTC handelt es sich um eine evidenzbasierte Interventionsstrategie, die konsequent theoretisch fundiert ist, empirische Erkenntnisse einbindet und qualitätsgesichert umgesetzt wird. Ziel ist es, die Kommunen zu befähigen, eine lokal passgenaue Strategie zur Prävention psychosozialer Probleme bei Heranwachsenden zu entwickeln. CTC versucht dabei, die Anforderungen an Evidenzbasierung und Partizipation zu verbinden, um eine weitestmögliche Akzeptanz eines wissenschaftsbasierten Vorgehens zu ermöglichen. Die vor knapp 15 Jahren nach Deutschland transferierte und strukturell sowie kultursensibel angepasste systemische Intervention ist inzwischen in mehreren Bundesländern verbreitet.

Die vom Bundesministerium für Bildung und Forschung (BMBF) geförderte Studie „Effektivität des kommunalen Präventionssystems Communities That Care – CTC – EFF“ (2021–2023) untersucht in einem „cluster non-randomized controlled trial“ (ClNRCT), ob sich die Effekte der Originalstudie replizieren lassen. Einbezogen sind 3 Bundesländer mit insgesamt 38 CTC-Kommunen und Vergleichskommunen unterschiedlicher Größe und Sozialstruktur [47, 48]. Die eingesetzten Erhebungsinstrumente wurden in mehreren Schritten an den deutschen Kontext angepasst und haben sich nach ersten Auswertungen als valide erwiesen. Erste Auswertungen zeigen auch für CTC in Deutschland, dass sich die Wahrscheinlichkeit, dass Kommunen PGF wissenschaftsbasiert umsetzen, durch eine koordinierte Zusammenarbeit (26-fach) und eine bedarfssensible Planung (20-fach) erhöht. In Kommunen, die über eine hohe Bereitschaft und angemessene Ressourcen für PGF verfügen, haben Kinder und Jugendliche ein 3,5-fach geringeres Risiko, Alkohol zu konsumieren, sowie ein 2,5-fach geringeres Risiko zu rauchen. Die längsschnittlichen Ergebnisse werden zeigen, ob CTC auch in Deutschland die dritte Ebene der Evidenz [10] erreicht und reif für eine flächendeckende Dissemination ist.

Ergänzend zum kommunalen Ansatz wurde das CTC-Konzept für eine systemische und evidenzbasierte Entwicklung der PGF in Schulen adaptiert (Schools That Care – STC, https://schoolsthat.care/). In den nächsten Jahren ist vorgesehen, dieses Konzept in mehreren Bundesländern mit begleitender Evaluation umzusetzen. In diesem Zusammenhang wird die Grüne Liste Prävention um verhaltensbezogene Intervention sowie um die Handlungsfelder Ernährung und Bewegung erweitert. Damit werden auch in Deutschland Entwicklungen vollzogen, die bereits in den USA mit der Öffnung und Weiterentwicklung des Evidenzregisters Blueprints und der Ausweitung von CTC auf andere Handlungsfelder erfolgten.

### Infobox 1 Evidenzbasierte Maßnahmen: die „Grüne Liste Prävention“

Die Grüne Liste Prävention ist ein kostenloses und frei zugängliches Online-Register (www.gruene-liste-praevention.de), die Praktiker:innen, Entscheidungsträger:innen und Wissenschaftler:innen einen Überblick über evidenzbasierte Programme zur psychosozialen Gesundheit von Kindern und Jugendlichen gibt. Das 2011 vom Landespräventionsrat Niedersachsen (LPR) im Rahmen von CTC aufgebaute Evidenzregister ist inzwischen über den CTC-Kontext hinaus bekannt. Die „Grüne Liste Prävention“ wird im „Memorandum Evidenzbasierte Prävention und Gesundheitsförderung“ der Bundeszentrale für gesundheitliche Aufklärung (BZgA; [10]) als „Best-evidence“-Datenbank eingeordnet. Über 90 % der Programme sind nach Kriterien der Gesetzlichen Krankenversicherung (GKV) förderfähig (Stand: 03/2023; [49]).

Die Maßnahmen werden systematisch bewertet und entsprechend ihren Ergebnissen in *3 Evidenzgrade* eingestuft: (1) Effektivität theoretisch gut begründet, (2) Effektivität wahrscheinlich und (3) Effektivität nachgewiesen. Die Einstufung erfolgt anhand von definierten Bewertungs- und Aufnahmekriterien in den Bereichen Konzept‑, Umsetzungs- und Evaluationsqualität [12].

Aktuell sind in der Grünen Liste Prävention 100 PGF-Maßnahmen gelistet (Stand: 01/2023), wobei einige mehrere Zielgruppen, Lebenswelten und Themen gleichzeitig fokussieren [49]. Primäre Zielgruppen sind Säuglinge und Kleinkinder (*n* = 23 Maßnahmen), 4‑ bis 6‑Jährige (*n* = 33), 6‑ bis 10-Jährige (*n* = 45) und 11- bis 18-Jährige (*n* = 60). Lebenswelten umfassen als zentrale Sozialisationsinstanz die Schule (*n* = 61 Maßnahmen), Kita (*n* = 23), Krippe (*n* = 5) und die Familie (*n* = 28). Ausschließlich verhaltensbezogen sind 92 Programme, 7 haben einen verhaltensbezogenen Schwerpunkt mit verhältnisbezogenem Anteil, und eine Maßnahme ist verhältnispräventiv. Mehr als die Hälfte sind Sozial- und/oder Lebenskompetenztrainings (*n* = 61).

Weitere häufige Umsetzungsformen bilden Trainings der Erziehungsberechtigten (*n* = 25) und frühkindliche Interventionen (*n* = 14).

Zentrale Themen sind Gewalt (*n* = 64 Maßnahmen, z. B. Mobbing, Delinquenz, sexueller Missbrauch), substanzgebundene (*n* = 45, z. B. Rauchen, Alkohol, Drogen) und substanzungebundene Abhängigkeit (*n* = 3, z. B. Internetabhängigkeit, Glücksspiel), psychische Gesundheit (*n* = 34, z. B. Depression, Ängste, Stressbewältigung) sowie reduzierte Alltagskompetenzen (*n* = 32, z. B. Störung der sozialen Interaktion, Problemlösefähigkeiten).

### Infobox 2 Anforderungen an eine wirksame Prävention und Gesundheitsförderung (PGF) und Communities That Care (CTC)

Soll PGF erfolgreich umgesetzt werden und wirksam sein, muss sie zentrale Qualitätskriterien erfüllen [17]. Nach den Good-Practice-Kriterien einer soziallagenbezogenen Gesundheitsförderung [18] sind 12 Kriterien zentral: Zielgruppenbezug, Konzeption (logisch, theoretisch gut begründet), Setting-Ansatz, Empowerment, Partizipation, niederschwellige Arbeitsweise, Multiplikator:innenkonzept, Nachhaltigkeit, integriertes Handeln, Qualitätsmanagement, Dokumentation und Evaluation sowie Belege für Wirksamkeit und Kosten. Nach unserer Analyse erreicht das CTC-Präventionssystem jeweils die höchste Stufe dieser Anforderungen.

In dem Memorandum zur evidenzbasierten PGF [10] formulieren de Bock, Dietrich und Rehfuss allgemeine Prinzipien und Umsetzungsfaktoren von Evidenzbasierung sowie 3 Stufen evidenzbasierter Interventionen. Diese greifen die obigen Kriterien z. T. auf, gehen aber über diese hinaus. Tab. 1 zeigt, wie CTC die Anforderungen realisiert. Deutlich wird, dass CTC alle Anforderungen an Evidenzbasierung erfüllt.

**Table 1:** 

Anforderungen [10]		Umsetzung in CTC
*Allgemeine Prinzipien von Evidenzbasierung STIIP*		
Systematik	Die evidenzbasierte Entscheidung basiert auf einer systematischen Sichtung der Studienlage zur Wirksamkeit der Maßnahme	Die KK greift auf ein Evidenzregister mit systematisch aufbereiteter Evidenz zurück (Grüne Liste Prävention)
Transparenz im Umgang mit Unsicherheiten	Der Entscheidungsfindungsprozess ist explizit gestaltet. Unsicherheiten in der Evidenz zur Wirksamkeit werden über Evidenzgrade differenziert dargestellt	(1) Die KK führt einen vorstrukturierten Prozess der Entscheidungsfindung durch (2) Das Register legt Unsicherheiten in der Evidenz mittels Evidenzgrade offen
Integration und Partizipation	An der Entscheidungsfindung sind die Betroffenen und möglichst alle Stakeholder beteiligt	Die KK beteiligt alle wesentlichen lokalen Stakeholder. Sie entscheidet selbstständig, welche Maßnahmen durchgeführt werden. Dabei wird sie durch die CTC-Prozessbegleitung unterstützt
Umgang mit Interessenkonflikten	Unterschiedliche Motivationen und Interessen müssen in Einklang gebracht werden, Interessenkonflikte sind offenzulegen und sollten nicht systematisch evidenzbasierte Entscheidungen verzerren	(1) Die vorstrukturierte Prozessgestaltung mit intensiver Begleitung minimiert Verzerrungen in der Entscheidungsfindung durch die KK (2) Das Evidenzregister legt mögliche Interessenkonflikte nach wissenschaftlichen Maßstäben offen
Strukturierter, reflektierter Prozess	5 Schritte zur Evidenzbasierung: (1) klare Fragestellung, (2) Suche nach bester verfügbarer Evidenz, (3) kritische Prüfung wissenschaftlicher Erkenntnisse hinsichtlich Glaubwürdigkeit und Relevanz, (4) Anwendung der Evidenz, (5) Bewertung der Umsetzung	(1) Die KK bewertet die Maßnahmen hinsichtlich der lokalen Anwendbarkeit und Umsetzbarkeit. Die klare Fragestellung ergibt sich aus der Ergebnisanalyse der Kinder- und Jugendbefragung: Auswahl an zu beeinflussenden RF/SF durch die KK (2) Das Evidenzregister gibt Aussagen zur kritischen strukturierten Prüfung der Evidenz
*Umsetzungsfaktoren von Evidenzbasierung TIKKA*		
Theorie	Public-Health-Maßnahmen müssen theoretisch fundiert sein bezüglich ihrer Wirkmechanismen	Das CTC-System basiert auf (1) einem RF/SF-Modell, (2) einem Sozialentwicklungsmodell, (3) einer Theory of Change, (4) theoriebasierten Programmen im Evidenzregister
Interdisziplinarität	Die Wirksamkeit und Umsetzbarkeit von Public-Health-Maßnahmen hängen davon ab, ob ihre Entwicklung und Umsetzung interdisziplinär erfolgen	(1) Das CTC-System ist interdisziplinär entwickelt worden (2) Die KK ist intersektoral und interdisziplinär zusammengesetzt
Kontextabhängigkeit und Komplexität	Komplexe Maßnahmen umfassen Einzelelemente verschiedener Ebenen; Wechselwirkungen mit dem Kontext müssen bedacht, förderliche/hinderliche Faktoren identifiziert werden. Hilfreich ist ein logisches Modell	(1) Das CTC-System ist eine komplexe systemische Mehrebenen-Intervention (2) Dem CTC-System liegt ein logisches Modell zugrunde, in dem Kontextabhängigkeiten berücksichtigt sind
Allgemeine gesellschaftliche Aspekte	Integration von Aspekten in Entscheidungsprozessen, z. B. in Evidence-to-Decision-Framework: Nutzen/Schaden, Akzeptanz, Machbarkeit, Kosten, Auswirkungen auf gesundheitliche Chancengleichheit und Umwelt	Der CTC-Prozess lässt den lokalen CTC-Akteur:innen ausreichend Spielräume, um Aspekte wie Akzeptanz, Machbarkeit, Förderung von Chancengleichheit bei der Priorisierung von RF/SF sowie bei der Auswahl und Implementation von evidenzbasierten Maßnahmen zu berücksichtigen
*Evidenzbasierte Interventionen *(Definitionen s. [10])		
Wirksamkeit unter Studienbedingungen (Efficacy)		Mit der Community Youth Development Study liegt eine Efficacy-Studie vor
Wirksamkeit unter Alltagsbedingungen (Effectiveness)		Effectiveness-Studien zu CTC liegen vor (Australien, USA, Niederlande). In Deutschland wird seit 2020 die Wirksamkeit überprüft
Wirksamkeit in unterschiedlichen Kontexten für flächendeckende Umsetzung (Dissemination)		Eine international vergleichende und eine sozialraumvergleichende Studie zur Wirksamkeit von CTC stehen noch aus

*KK* kommunale Kooperationsgruppe; *RF/SF* Risiko- und Schutzfaktoren; *STIIP* Systematik, Transparenz im Umgang mit Unsicherheit, Integration und Partizipation, Umgang mit Interessenkonflikten sowie ein strukturierter, reflektierter Prozess; *TIKKA* Theorie, Interdisziplinarität, Kontextabhängigkeit und Komplexität sowie allgemeine gesellschaftliche Aspekte

## Footnotes

1 https://www.augsburg.de/umwelt-soziales/kinder-und-jugendliche/communities-that-care.
